# Pathological Cycle between Pain, Insomnia, and Anxiety in Women with Fibromyalgia and its Association with Disease Impact

**DOI:** 10.3390/biomedicines11010148

**Published:** 2023-01-06

**Authors:** Patricia Catalá, Lorena Gutiérrez, Carmen Écija, Cecilia Peñacoba

**Affiliations:** Department of Psychology, Rey Juan Carlos University, Avda. de Atenas s/n, 28922 Alcorcón (Madrid), Spain

**Keywords:** pain, anxiety, insomnia, fibromyalgia, mediation analysis, disability studies, behavioral medicine

## Abstract

Background: Pain, sleep disturbances, and mood disorders are considered common symptoms of fibromyalgia (FM). However, the interactions that are established between them and the implication that this has in the disease are not clear. The main objective of this study is to clarify the relationships established between insomnia, pain intensity and anxiety in women with FM. Additionally, the effect that the indicated pathological cycle between pain, insomnia and anxiety may have on the impact of the disease in these patients is explored. Methods: A total of 228 women diagnosed with FM participated in this study (mean age = 56.99 years, SD = 10.35). Measurements were conducted at two time points. Initially, the women completed self-report questionnaires about anxiety (The Hospital Anxiety and Depression Scale; HADS), sleep problems (The Insomnia Severity Index; ISI) and pain intensity (Brief Pain Inventory; BPI), and a week later, they answered questions about the impact of fibromyalgia (Fibromyalgia Impact Questionnaire- Revised; FIQ-R). For data analysis, models 4 and 6 of the Macro Process for SPSS were used. Results: Insomnia predicts higher levels of pain, which in turn predicts higher levels of anxiety, which in turn predicts a higher impact of fibromyalgia (B = 2.76, SE = 1.10, 95% CI = [0.79,5.11]). No significant results were found for the other interactions between the variables. Conclusions: Due to the clinical and scientific relevance of the insomnia–pain–anxiety pathological cycle and given the impact it has on FM, it is especially relevant to develop programs for patients with fibromyalgia based mainly on improving sleep quality.

## 1. Introduction

Fibromyalgia (FM) is considered a chronic disease without a well-established etiology, characterized mainly by widespread pain, fatigue, sleep and/or mood disorders [1]. This syndrome has a considerable impact on symptomatology and on activities of daily living in patients who suffer from it [2,3]. Although pain has been the symptom to which the greatest research and therapeutic efforts have been devoted, more than 90% of FM patients report sleep problems [4,5]. It has been observed that patients with significant sleep disturbances have lower pain thresholds [6,7] and perceive a greater severity of the disease [8]. In turn, chronic pain has been shown to favor the onset of sleep disturbances [9,10]. In this way, a bidirectional relationship is established between sleep disorders and chronic pain that generates a dangerous vicious circle in terms of health [11,12,13,14].

Another frequent comorbidity associated with chronic pain and sleep disorders is mood disorders [15]. It is known to all how chronic pain and anxiety influence each other. It has been proven that anxiety is associated with a greater severity of pain [16,17], and in turn, the presence of chronic pain causes higher levels of anxiety. Furthermore, in FM, sleep disorders have been associated with higher levels of anxiety [5]. Thus, sleep disorders, chronic pain, and mood disorders (particularly anxiety) appear to be related from a clinical perspective. These interactions could be related to the malfunction of the central nervous system due to FM [18]. Furthermore, it is interesting to note that the brain areas and neurotransmitter pathways involved in pain modulation, the sleep–wake cycle, and anxiety overlap [7,19].

A recent case study analyzing the pathological cycle of pain–insomnia–anxiety in FM patients [18] suggests that the direction is stronger from sleep disturbances towards chronic pain. Thus, it is pointed out that the loop would be preferentially oriented in the direction of insomnia to pain and anxiety. However, these results obtained by Mory et al. [18] should be treated with caution since they used a small sample (27 participants). In this context, given the inconsistency in the results regarding the functional relationship (antecedent and consequent) between pain, insomnia and anxiety and the need to analyze these models in larger samples, the present study is proposed to advance this research question. In addition, to our knowledge, no studies have been carried out that analyze the impact of the previous pathological circles on the impact of the disease. For this reason, and given the clinical and scientific relevance that this statement would imply, the main objective of this study is to clarify the relationships established between insomnia, pain intensity and anxiety in women with fibromyalgia. Additionally, the present investigation aims to explore the effect that the indicated pathological cycle between pain, insomnia and anxiety can have on the impact of the disease in these patients. This last objective is especially relevant in patients with fibromyalgia, since compared other chronic pain disorders, these patients experience great functional limitations.

## 2. Materials and Methods

### 2.1. Sample

The participants of the present study were 228 women diagnosed with fibromyalgia according to the criteria of the American College of Rheumatology [20,21]. Seventy-eight percent of the women had been diagnosed in a rheumatology service, 7% in primary care, and 15% in other services (traumatology, rehabilitation, neurology, internal medicine, or pain clinic). The following inclusion criteria were followed: having a diagnosis of FM, being over 18 years of age and giving written consent to participate in the research. To recruit the sample, a psychologist from the research team contacted different mutual aid associations for fibromyalgia patients in Spain, specifically in Madrid, Ciudad Real, Albacete, Guadalajara and Toledo. In Spain, the vast majority of patients with FM belong to an association, since it represents significant savings for patients and health systems [22].

A total of 268 women with fibromyalgia agreed to participate in the study and met our inclusion criteria. The patients were distributed in groups of 8–10. To facilitate the participation of the patients, the research team (two of its members) traveled to the place where the participants lived. Therefore, the evaluations were carried out at the associations to which the patients belonged. The patients were evaluated twice. The evaluation sessions lasted approximately 30 min. Self-report questionnaires (paper-and-pencil form) were administered to each of them. In the first evaluation, the patients were informed about the research and those who agreed to participate signed the informed consent form. Then, the participants answered the insomnia, severe pain and anxiety questionnaires. In a second evaluation, a week later, the patients were asked about the impact of fibromyalgia (self-report). In order to connect the data in the two stages of the study, a code was assigned to each of the participants. In both assessments, the researchers were present to clarify any question that caused doubts in the completion of the self-report.

The final sample comprised a total of 228 patients of the 268 initially interested in participating (28 did not attend the scheduled assessment appointment, 9 questionnaires were left blank, and 3 questionnaires contained missing data that could not be retrieved because participants could no longer be contacted).

The study was carried out between January 2017 and June 2018. This multicenter study was conducted according to the guidelines of the Declaration of Helsinki and approved by the University Ethics Committee of the Universidad Rey Juan Carlos (Reference 160520165916, date of approval 9 June 2016).

### 2.2. Measures

*Insomnia*–The Insomnia Severity Index (ISI) was used to assess insomnia [23]. The content of the ISI corresponds in part to the DSM-IV diagnostic criteria for insomnia. The questionnaire is made up of 7 items with a Likert-type response format of 5 points (0 to 4, where 0 indicates no problem and 4 corresponds to a very serious problem), yielding a score that ranges from 0 to 28. Higher scores represent poorer subjective sleep quality. The internal consistency in this sample was 0.92.

*Pain severity*–The pain severity scores were obtained by averaging the maximum, minimum and overall pain severity during the last 7 days, together with the pain severity rating at the time of assessment [24]. All four items to assess pain severity were extracted from the Brief Pain Inventory [25]. Pain severity measures were based on retrospective reports, with the exception of the measure of current pain severity at the time of assessment. All of these measures were rated with an 11-point numerical rating scale (0 = “no pain” and 10 = “the worst pain you can imagine”) [26]. The internal consistency of the four items in this sample was 0.91.

*Fibromyalgia impact*–The Spanish adaptation of the Fibromyalgia Impact Questionnaire—Revised was used to measure the impact of fibromyalgia on functioning [27]. The total score was used. It consists of 21 items with an 11-point Likert response (from 0 to 10) format that evaluates three associated domains: physical function, overall impact and symptoms. This scale provides a total score (ranging from 0 to 100), where higher scores represent higher perceived impact of fibromyalgia on functioning. The total FIQ-R score is more frequently used to reduce the number of statistical comparisons [28]. The Cronbach’s alpha in the present study was 0.88.

*Anxiety*–The anxiety subscale of the Spanish version of the Hospital Anxiety and Depression Scale (HADS) [29] was used. This brief instrument has been widely used to measure the possible presence of states of anxiety and depression in non-psychiatric outpatient medical clinic settings. The anxiety subscale consists of 7 items, with a 4-point Likert response scale (from 0 = never to 3 = always). Scores less than eight indicate no anxiety symptoms. Scores between eight and thirteen indicate mild anxiety, scores between fourteen and twenty indicate moderate anxiety, and scores greater than twenty indicate symptoms of severe anxiety. The Cronbach’s alpha in the present study was 0.81.

*Socio-Demographic and clinical data*–An ad hoc questionnaire was used to evaluate age, educational level, employment status, and marital status. Regarding clinical variables, the duration of fibromyalgia was recorded.

### 2.3. Statistical Analysis

The SPSS 22 statistical package [30] was used to perform the analyses. Descriptive analysis, internal consistency analysis (Cronbach’s alpha coefficients) and Pearson correlation analysis were performed. In addition, means, standard deviations, and median ranges were used for continuous variables. Categorical data were presented as numbers and percentages. The statistical significance level for all tests was set at a *p* value less than 0.05.

For the analysis of simple mediation (model 4) and multiple serial mediation (Model 6; mediation of several mediators that go one after the other), applying two significant mediators, SPSS macro PROCESS was used. As Hayes recommended [31], the regression/trajectory coefficients were all in a non-standardized form since standardized coefficients generally do not have a useful substantive interpretation. First, as shown in Table 1, six different causal models were tested. Secondly, and taking into account the significant simple mediation models, its effect on the impact of the disease was verified. Model fit was also examined using the following criteria: a chi-square/df of ≤ 2, a *p*-value of > 0.05, and an approximation of root mean square error of < 0.06 [32]. In addition, to increase the robustness of the results, confidence intervals (CI) generated from bootstrapping effect estimation techniques were used. For a significant mediator effect, the CI limits did not include the value 0 [33,34]. A total of 5000 bootstrap resamples were used to generate bias-corrected 95% CIs for the indirect effect.

## 3. Results

### 3.1. Sample Characteristics

The mean age of the women was 56.99 years (SD = 10.35). In relation to education, 6% of the women had completed university, 26.6% secondary education, 51.3% primary education and 15.6% knew how to read and write. The majority of the women were married or in a stable relationship (78.1%), 6% were single and 15.9% were divorced or widowed. Seventy-eight percent of the women were housewives. Participants had a fibromyalgia diagnosis for an average of 13.02 years (SD = 7.21, range 1 to 46 years).

### 3.2. Means, Standard Deviations, Minimum, Maximum and Pearson Correlations between the Study Variables

Table 2 shows the means, standard deviations and Pearson correlations between the study variables. As shown in Table 1, significant positive relationships are observed between all of the study variables (all *p* > 0.05).

### 3.3. Testing of Mediation Models

Table 3 shows the mediation indices for each of the tested models (see Table 1). Taking into account the fact that there is a significant mediating effect when the CI limits do not include the value 0, the results show that model 1 was the only significant one (B = 0.11, SE = 0.007, 95% CI = [0.001, 0.036]). That is, insomnia predicts higher levels of pain, and this, in turn, predicts higher levels of anxiety.

### 3.4. Effect of Insomnia on the Impact of Fibromyalgia through Pain Severity and Anxiety

**Figure 1:**
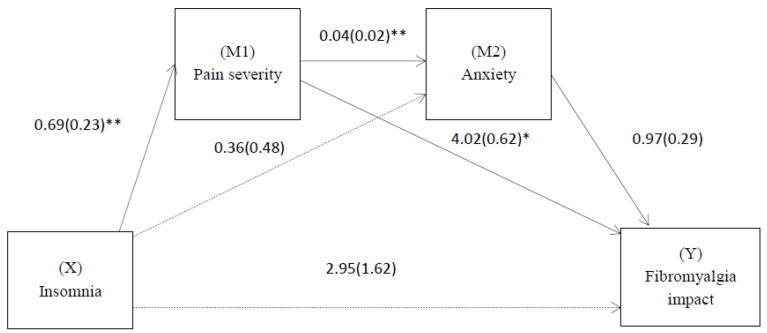
Path diagram illustrating direct effects and causal paths linking pain severity with the fibromyalgia impact. Notes: Multiple mediation analysis in series with the fibromyalgia impact as the dependent variable, insomnia as the independent variable and pain severity and anxiety as the first and second mediators. Values are non-standardized regression coefficients (SE in parentheses) and associated *p* values (* *p* < 0.05, ** *p* < 0.01). Association in brackets = direct effect (controlling for indirect effects). Solid lines indicate important pathways and dashed lines indicate non-significant pathways.

Based on the results of the mediation models, only the effect of model 1 on the impact of fibromyalgia was tested. A significant indirect effect of insomnia on the impact of fibromyalgia was observed through pain severity and anxiety (B = 2.76, SE = 1.10, 95% CI = [0.79, 5.11]) (Figure 1). That is, greater insomnia predicted greater pain severity, which predicted greater anxiety, which in turn predicted a greater impact of fibromyalgia. There was also a significant simple indirect effect of insomnia on the impact of fibromyalgia via pain severity (B = 0.28, SE = 0.21, 95% CI = [0.01, 0.81]). In contrast, no mediating effect of anxiety was observed (B = −0.35, SE = 0.42, 95% CI = [−0.43, 1.26]). There was also no direct effect (B = 2.95, SE = 1.62, t = 1.82, 95% CI = [−0.25, −6.16], *p* = 0.10) of the predictor on the impact of fibromyalgia. Figure 1 provides additional details on this analysis. The contrast test, when comparing the indirect effects, showed that the indirect effect through the two mediators in series was higher than the indirect effect through pain severity as a mediator (B = −2.48, SE = 1.04, 95% CI = [0.65, 4.76]).

## 4. Discussion

The main objective of this study was to elucidate the associations established between insomnia, pain severity and anxiety, thus determining the possible impact of the interaction of these variables on the perception of the disease in women with fibromyalgia. One of the most important findings obtained was verifying that, in line with what is suggested by the latest research [15,18], the pain–insomnia–anxiety pathological cycle is preferably oriented in the direction from insomnia to pain and, secondarily, from pain to anxiety. These results have important clinical implications, since insomnia can cause higher levels of pain and increased pain can induce higher levels of anxiety. This result is especially interesting, since it indicates that insomnia has effects on anxiety when the patient is in pain. As it is a pathology characterized by the presence of chronic pain, it is possible that this mediating effect goes unnoticed. This fact is especially relevant in the design of the intervention, since if the patient with sleep problems does not manage to perceive an improvement in pain levels first, it is very possible that anxiety levels will not be reduced.

The exposed mediation model adds that the insomnia–pain–anxiety pathological cycle, in turn, predicts a greater impact of the disease. The literature is consistent in the repercussions that presenting this symptomatology has on the impact of the disease [3,35]. Previous studies have revealed that higher levels of pain and anxiety or sleep difficulties are independently associated with the impact of the disease, being especially relevant in functional limitations and in the interference of the disease with daily life [3,36,37,38]. However, there is limited evidence examining the impact on the disease of the interaction of these variables [39], which coexist in many of these patients. Despite the fact that there are studies that analyze the reciprocal relationships between sleep disturbances, pain, and anxiety in fibromyalgia [18], to date, no conclusive results have been found regarding the impact of these interactions on the manifestations of FM. Thus, the results presented here shed clarity on this question. This study shows as a novelty, in addition to the direction of the potential link between insomnia, chronic pain and anxiety, the impact that this relationship has on functional limitations in patients with fibromyalgia. Therefore, it would be advisable to promote good sleep hygiene to allow the improvement of the other components. A current review reports that cognitive–behavioral therapy is the main recommended treatment option for patients with sleep disorders and chronic pain [10]. The treatment effect with this type of therapy has been shown to last longer compared to pharmacological options, which generally stop working once the medication is discontinued [10,40]. Specifically, long-lasting effects of up to 18 months after treatment have been observed [41].

On the other hand, in the present study, the simple indirect effect of insomnia on the impact of fibromyalgia through pain was significant. These results are consistent with research using the time series method with daily monitoring in patients with fibromyalgia. The findings of these studies show that after a night of poor sleep, pain intensity increases [42] and, moreover, that greater sleep interruption predicts higher levels of pain the following day [43]. These results further support the hypothesis that poor sleep quality is a pathogenic stimulus for fibromyalgia.

Certain limitations of this study should be mentioned. First, the sample is made up only of women with fibromyalgia, which does not allow the results to be generalized to other populations with chronic pain. Second, comorbidity was not controlled, so the results should be taken with caution. In addition, it should also be taken into account that there is no universally accepted definition of poor sleep and there is a wide variety of self-report questionnaires to assess sleep quality, which makes comparison with other studies difficult. Although the questionnaire used in this study was the one used by most of the articles, it is important to standardize the evaluation of sleep.

## 5. Conclusions

Due to the clinical and scientific relevance of the pathological cycle of insomnia–pain–anxiety and taking into account the findings obtained in this study, it is especially relevant to develop programs for patients with fibromyalgia based mainly on improving sleep quality. The findings presented show that insomnia is related to anxiety only if patients also have pain. Taking into account the fact that pain is one of the central symptoms of fibromyalgia, it is not uncommon for the three components of insomnia–pain–anxiety to appear in most patients diagnosed with the disease.

## Figures and Tables

**Table 1:** Causal models tested.

Model	Predictor (X)	Mediator	Outcome (Y)
1	Insomnia	Pain severity	Anxiety
2	Insomnia	Anxiety	Pain severity
3	Pain severity	Insomnia	Anxiety
4	Pain severity	Anxiety	Insomnia
5	Anxiety	Pain severity	Insomnia
6	Anxiety	Insomnia	Pain severity

**Table 2:** Means, standard deviations, and Pearson correlations between study variables.

	Mean (SD)	Range Sample	2	3	4
1. Pain severity	7.23 (1.49)	1–10	0.22 **	0.34 **	0.58 **
2. Anxiety	12.17 (3.69)	4–21		0.23 **	0.37 **
3. Insomnia	8.85 (2.35)	0–11			0.58 **
4. Fibromyalgia impact	75.21 (18.42)	6–110			

*** *p* < 0.001, ** *p* < 0.01, * *p* < 0.05.

**Table 3:** The mediation indices for the tested models.

Model	Effect	SE	Boot LLCI	Boot LLCI
1	0.011	0.007	0.001	0.036
2	0.066	0.053	−0.019	0.189
3	0.036	0.048	−0.040	0.153
4	0.006	0.010	−0.012	0.018
5	0.300	0.184	−0.001	0.694
6	0.041	0.012	−0.002	0.044

## Data Availability

The data presented in this study are available on request from the corresponding author.
